# Effluent quality soft sensor for wastewater treatment plant with ensemble sparse learning-based online next generation reservoir computing

**DOI:** 10.1016/j.wroa.2024.100276

**Published:** 2024-11-10

**Authors:** Gang Fang, Daoping Huang, Zhiying Wu, Yan Chen, Yan Li, Yiqi Liu

**Affiliations:** a Key Laboratory of Autonomous Systems and Networked Control, Ministry of Education, School of Automation Science &Engineering, South China University of Technology, Guangzhou, 510640, China; b Unmanned Aerial Vehicle Systems Engineering Technology Research Center of Guangdong, School of Automation Science &Engineering, South China University of Technology, Guangzhou, 510640, China; c Centre for Artificial Intelligence & Robotics, Hong Kong Institute of Science & Innovation, Chinese Academy of Sciences, Hong Kong; d School of Data Science and Information Engineering, Guizhou Minzu University, Guiyang 550025, China

**Keywords:** Soft sensor, Effluent variable prediction, Next generation reservoir computing, Sparse identification, Wastewater treatment plants

## Abstract

Real-time monitoring of key quality variables is essential and crucial for stable and safe operations of wastewater treatment plants (WWTPs). Next generation reservoir computing (NG-RC) has recently garnered significant attention in quality prediction, such as COD and BOD, as an effective alternative to traditional reservoir computing (RC), then is able to act as a data-driven soft sensor to twin a hardware sensor for quality variable measurements. Unlike RC, NG-RC does not require random sampling matrices to define the weights of recurrent neural networks and has fewer hyperparameters. However, NG-RC is usually used online but trained offline, thus leading to model degradation under dynamic scenarios. This paper proposes a sparse online NG-RC approach to meet the real-time requirements of WWTPs and mitigate the impact of measurement noise on the model. First, inspired by the Woodbury matrix identity, an incremental strategy is designed, using sequentially arriving data blocks to learn the output weights of NG-RC online. Then, an ensemble sparse strategy is combined to alleviate overfitting issues of the prediction model. Moreover, a soft sensor based on the ensemble sparse online NG-RC is developed to perform real-time prediction of quality indicators in wastewater treatment processes. Finally, two datasets from actual WWTPs are used to validate the effectiveness of the proposed model.

## Highlights

• A novel soft sensor method is proposed to enable multi-variable prediction.
• An incremental strategy is designed to implement online learning for the NG-RC model.
• An ensemble sparse strategy is combined to mitigate the overfitting issues of the NG-RC model.
• The methodology is validated through two wastewater treatment plant datasets with different patterns.

## Graphical abstract

**Figure:**
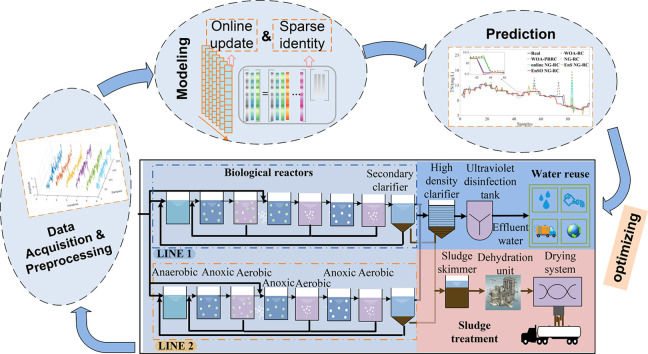

## 1 Introduction

In wastewater treatment processes, proper monitoring of key quality variables, such as biochemical oxygen demand (BOD) and chemical oxygen demand (COD), is essential for the stable and safe operation of systems (Newhart et al., 2019; Muniappan et al., 2023). However, due to prohibitive costs or harsh on-site condition such as strong acids and abrasion, these critical quality variables cannot be measured directly (Li et al., 2024). In contrast, certain process variables such as temperature, flow rate, and pH are relatively easy to measure (Reynaert et al., 2023). Consequently, soft sensors and digital twin techniques can monitor key quality variables by establishing relationships between these easily measurable variables and the difficult-to-measure variables. Digital twin can be understood as a digital representation of a physical entity and the twinned information can be fed back into early inspection, physical sensor maintenance and planning for replacement (A.J. Wang et al., 2024). Soft sensors can effectively predict effluent quality indicators and are able to serve as reliable backups during hardware sensor maintenance (Zhu et al., 2022).

Recurrent neural networks (RNNs) and long short-term memory (LSTM) have been widely applied in predicting effluent quality indicators of WWTPs due to their strong capability in handling long-term dependencies in time series data. Also, Wongburi et al. utilized RNNs to predict effluent BOD (Wongburi and Park, 2023). Yu et al. developed LSTM-based soft sensors using low-cost sensor datasets, achieving the prediction of effluent COD (Yu et al., 2023). Reservoir computing (RC), a variant of RNNs, has garnered significant attention in recent years within the field of soft sensor modeling (Kim et al., 2020). Compared to traditional RNNs and LSTM, RC only requires training of the output layer weights rather than the entire weight network, substantially reducing training time (T.C. Wang et al., 2024). This makes it highly suitable for WWTPs that require real-time monitoring. For instance, Liu et al. utilized an improved sparrow search algorithm to optimize the hyperparameters of RC, achieving predictions of effluent BOD and COD in wastewater treatment plants (Liu et al., 2024). Moreover, WWTPs typically involve complex nonlinear and time-varying characteristics. Reservoir computing excels in handling such systems by enabling more accurate modeling (Lukoševičius and Jaeger, 2009). For example, Yang et al. proposed an evolving deep delay RC, which achieved precise prediction of effluent ammonia nitrogen concentration in WWTPs (Yang et al., 2023). However, RC introduces some randomness in the results when using random matrices to define the internal and input weights of the reservoir. This randomness may lead to unacceptable outcomes, and there is little effective guidance to address this issue (Tanaka et al., 2019). Furthermore, the performance of RC is affected by several important meta-parameters, and optimizing these parameters adds extra computational time. In light of these challenges, Gauthier et al. investigated the relationship between RC and nonlinear vector autoregression, leading to the development of next generation reservoir computing (NG-RC) (Gauthier et al., 2021).

NG-RC simplifies the structure of RC by replacing the reservoir with feature vectors. Unlike RC, NG-RC does not require random matrices to define the input and internal weights of the reservoir, and fewer hyperparameters are needed. Recently, NG-RC has been widely applied in time series prediction and dynamic forecasting of chaotic systems. Brucke et al. utilized NG-RC for day-ahead energy demand forecasting (Brucke et al., 2024), Ratas et al. employed NG-RC to predict the dynamics of chaotic systems (Ratas and Pyragas, 2024), and Chepuri et al. proposed a hybrid method, RC—NGRC, for time series forecasting (Chepuri et al., 2024). NG-RC achieved similar prediction accuracy in these studies compared to RC with fewer computational resources. However, existing NG-RC models still have limitations. Most NG-RC models are designed and analyzed using batch learning algorithms, which cannot adapt to real-time changes in wastewater treatment processes. In addition, the output weights trained by NG-RC are susceptible to noise interference, thus usually leading to model overfitting. Online learning is a widely adopted approach for enhancing the real-time performance and adaptability of models, while sparse learning can effectively address overfitting issues in model training (Liu and Qin, 2023). Therefore, exploring sparse online NG-RC suitable for wastewater treatment processes is important for efficient data mining and soft sensor modeling.

Many researchers have devoted to online learning and sparse learning for RC and NG-RC. For instance, Schwedersky et al. proposed a recursive least squares (RLS) algorithm with directional forgetting factors for online identification of nonlinear systems, enabling online updates of RC (Schwedersky et al., 2022). Chen et al. introduced a matrix inversion lemma to develop an online RC algorithm without additional hyperparameters, successfully predicting robotic joint velocities (Chen et al., 2024). However, few studies have addressed the issue of noise interference in the context of online updating of RC algorithms. To address overfitting issues caused by noise from the measurements, Yang et al. applied regularization techniques and proposed a sparse RLS algorithm for estimating output weights of RC, predicting the concentration of ammonia nitrogen in the effluent (Yang et al., 2019). Zhang et al. successfully reconstructed complex high-dimensional attractor basins using NG-RC after sparsifying the training data (Zhang and Cornelius, 2023). Nevertheless, these algorithms often introduce additional hyperparameters to achieve online updating of RC at the cost of increasing computational and design complexity. Therefore, existing algorithms usually fail to meet the real-time monitoring requirements of WWTPs. Furthermore, online updating and sparse learning of NG-RC remain an open problem in the wastewater treatment communities.

This paper proposes an ensemble sparse online NG-RC (EnSO NG-RC) model for real-time monitoring of key quality variables, such as COD and BOD, in WWTPs. First, inspired by the Woodbury matrix identity, a recursive formula is constructed using sequentially arriving data blocks to learn the output weights of NG-RC online. Subsequently, this paper employs the sparse identification theory of dynamic systems and ensemble learning to perform sparse identification on the online-updated output weights, aiming to address issues of limited data and model overfitting. Based on EnSO NG-RC, a soft sensor is then developed for real-time prediction of effluent variables in WWTPs. Finally, the model is validated using datasets from two actual WWTPs with diverse working conditions. Table 1 provides a list of abbreviations and their definitions.

**Table 1:** The glossary of abbreviations.

Abbreviations	Descriptions
AE	Absolute error
BOD	Biochemical oxygen demand
COD	Chemical oxygen demand
EnS NG-RC	Ensemble sparse NG-RC
EnSO NG-RC	Ensemble sparse online next generation reservoir computing
LASSO	Least absolute shrinkage and selection operator
LSTM	Long short-term memory
MAE	Mean absolute error
NG-RC	Next generation reservoir computing
PCC	Pearson correlation coefficient
RC	Reservoir computing
RLS	Recursive least squares
RMSE	Root mean square error
RMSSD	Root mean sum of squares of the diagonal
RNNs	Recurrent neural networks
SINDy	Sparse identification of nonlinear dynamics
SS	Suspended solids
STLS	Sequential thresholded least-squares
TN	Total nitrogen
TP	Total phosphorus
VIP	Variable importance in projection
WOA	Whale optimization algorithm
WOA-RC	Reservoir computing with hyper-parameters optimized by whale optimization algorithm
WOA-PRRC	Probabilistic regularization reservoir computing with hyper-parameters optimized by whale optimization algorithm
WWTPs	Wastewater treatment plants

## 2 Results and discussion

### 2.1 Experimental design

The real-time monitoring of key effluent indicators such as BOD, COD, and total nitrogen (TN) is crucial for the safe and stable operation of WWTPs. However, due to the high costs of online measurement instruments, high maintenance costs and harsh on-site environments, these critical quality variables are often difficult to measure online. Therefore, this study utilizes soft sensor technology to establish relationships between easily measurable process variables and key effluent indicators, enabling real-time monitoring of indicators like BOD and COD.

The proposed online NG-RC, ensemble sparse NG-RC (EnS NG-RC), and EnSO NG-RC methods were validated using data from two WWTPs: one located in Barcelona, Spain (Barcelona dataset) and another in Dongguan, China (Dongguan dataset). These methods were compared against NG-RC, WOA-RC, and WOA-PRRC approaches. WOA refers to the whale optimization algorithm, with WOA-RC being an RC model optimized using WOA for hyperparameter tuning. WOA-PRRC is an improved probabilistic regularization model based on WOA-RC. Detailed descriptions of WOA-RC and WOA-PRRC can be found in (Fang and Liu, 2024).

Prediction accuracy is a crucial factor to consider when developing soft sensors. To evaluate this accuracy, we employed the following metrics: mean absolute error (MAE), root mean square error (RMSE), Pearson correlation coefficient (PCC), and root mean sum of squares of the diagonal (RMSSD) (Fang and Liu, 2024). RMSSD describes the overall prediction error for multi-output models (Xiao et al., 2019) and is defined as follows:

(1) $$RMSSD=\sqrt{\frac{1}{n_{test}}\text{trace}\left({\left(Y-\hat{Y}\right)}^{T}\left(Y-\hat{Y}\right)\right)}$$

where $n_{test}$ is the number of test samples, Y and $\hat{Y}$ denote the real value and predicted value, trace(·) denote the trace of a matrix.

Based on the principle of experimental fairness, the EnSO NG-RC method is compared with other methods under identical hyperparameter settings. The experimental platform utilized is MATLAB version R2023b, operating on a Windows 10 environment with a 64-bit Intel Core i5–11,400 CPU at 2.60 GHz and 16.00 GB of RAM.

### 2.2 Performance of experiment I – A dataset from a Barcelona wastewater plant

#### 2.2.1 Background

The Barcelona dataset was acquired from a WWTP utilizing activated sludge technology, with measurement equipment that has undergone significant aging, as illustrated in Supplementary Materials Fig. S2. The data were sampled at one-day intervals over 527 days, including different weather and seasons. The plant has a wastewater treatment capacity of 35,000 m³/d. After preprocessing, 400 grab samples were utilized, and six process variables were selected as auxiliary variables through a combination of variable importance in projection (VIP), least absolute shrinkage and selection operator (LASSO), and domain expertise. The target variables for this study are effluent COD and BOD. The first 60 % of the samples were designated for training, while the remaining samples were allocated for testing purposes. Detailed information regarding the auxiliary variables can be found in (Fang and Liu, 2024).

#### 2.2.2 Experimental results and analysis

**Fig. 1:**
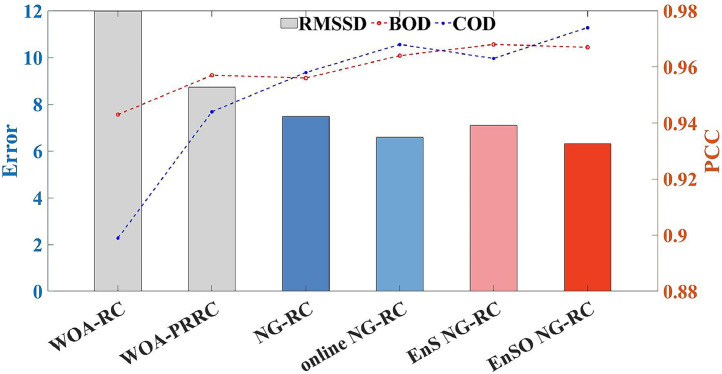
Prediction results of six models.

The hyperparameters for EnSO NG-RC were determined through trial and error, as shown in Supplementary Materials Table S1. Online NG-RC, EnS NG-RC, and NG-RC models utilize the same hyperparameters as EnSO NG-RC, where applicable. Table 2 presents the experimental results of the proposed online NG-RC, EnS NG-RC, and EnSO NG-RC models, along with comparative methods NG-RC, WOA-RC, and WOA-PRRC on the Barcelona dataset. Notably, the RMSSD for the three proposed soft sensors—online NG-RC, EnS NG-RC, and EnSO NG-RC, was reduced by 12.1 %, 5.28 %, and 15.8 %, respectively, compared to NG-RC. Furthermore, the RMSSD of NG-RC showed reductions of 37.5 % and 14.1 % compared to WOA-RC and WOA-PRRC, respectively. These results underscore the significant advantages of the NG-RC over traditional RC and improved RC, validating our choice of NG-RC as the foundational model for soft sensor modeling. To provide a clearer visualization of the prediction results, Fig. 1 was drawn. It visually demonstrates that the three proposed soft sensors not only have lower prediction errors compared to NG-RC but also show improvements in PCC of 0.837 %, 1.26 %, and 1.15 %, respectively.

**Table 2:** Comparison of prediction results of six models.

Model	RMSE		PCC		RMSSD
	BOD	COD	BOD	COD	
WOA-RC	1.891	11.820	0.943	0.899	11.970
WOA-PRRC	1.644	8.565	0.957	0.944	8.721
NG-RC	1.621	7.310	0.956	0.958	7.487
Online NG-RC	1.468	6.414	0.964	0.968	6.580
EnS NG-RC	1.462	6.940	0.968	0.963	7.092
EnSO NG-RC	**1.459**	**6.131**	**0.967**	**0.974**	**6.302**

**Fig. 2:**
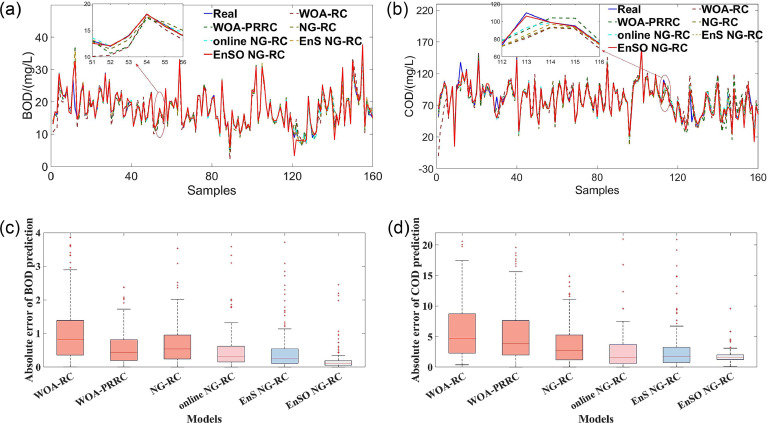
Fitting graphs of prediction results and box plots of absolute errors. (a) and (b) represent the fitting graphs for BOD and COD prediction results. (c) and (d) illustrate the box plots of absolute residual errors for BOD and COD predictions.

To comprehensively evaluate the model performance, we plotted fit plots of the prediction results and box plots of the absolute error (AE), as shown in Fig. 2. From Fig. 2(a) and (b), the online NG-RC and EnSO NG-RC models show superior tracking performance compared to NG-RC, especially at peak and trough positions. This indicates the effectiveness of our proposed online learning strategy in improving the real-time tracking capabilities of NG-RC. Fig. 2(c) and (d) reveal that the AE distribution of EnS NG-RC is more concentrated than that of NG-RC, suggesting that our ensemble sparse identification algorithm reduces the sensitivity of NG-RC to noise and improves the stability of prediction results. Furthermore, the EnSO NG-RC model, which combines online learning and ensemble sparsification, achieves the smallest RMSSD, validating the effectiveness of the EnSO strategy.

### 2.3 Performance of experiment II – A data set from a Dongguan wastewater plant

#### 2.3.1 Background

The dataset is derived from operational data of a WWTP in Dongguan, China, which uses a modified A^2^O process with a daily treatment capacity of 50,000 m³. The plant layout consists of four primary sections: gravel treatment, biological reactor, sludge treatment and water resource reuse. The biological reactor consists of one anaerobic tank, three anoxic tanks, three aerobic tanks and a secondary settling tank. The influent indicators at the anaerobic tank and the effluent at the secondary settling tank are critical. From the sensor data provided by the WWTP, this study selects influent COD, NH3-N, total phosphorus (TP), TN, suspended solids (SS), and flow rate Q as auxiliary variables. The target variables for real-time monitoring are effluent COD, TP, and TN, ensuring compliance with national water quality standards. The data were collected from a separate sewer system, with little impact from rainwater. Sensor data is sampled at 30-minute intervals, resulting in 48 samples collected daily for almost a month. Due to data loss from equipment maintenance and other factors, 480 samples of preprocessed data were used for the experiment. The first 80 % of the samples are designated as the training set, with the remaining samples allocated for testing.

#### 2.3.2 Experimental results and analysis

**Fig. 3:**
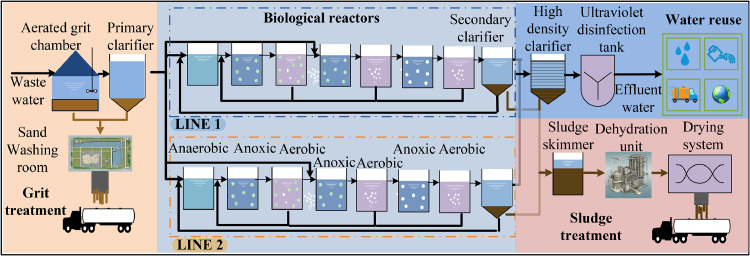
The overall layout of a Dongguan wastewater plant.

The hyperparameter selection for EnSO NG-RC is similar to that of the first experiment, as shown in Supplementary Materials Table S2. Experimental results for the proposed EnSO NG-RC and its comparative methods on the Dongguan dataset are presented in Supplementary Materials Table S3. Similar to the results of Experiment I, EnSO NG-RC achieved the lowest RMSSD, with a reduction of 58.1 % compared to NG-RC. These results not only validate the effectiveness of the proposed EnSO algorithm in improving the performance of NG-RC model, but also demonstrate a significant increase in prediction accuracy over traditional RC. Fig. 3

**Fig. 4:**
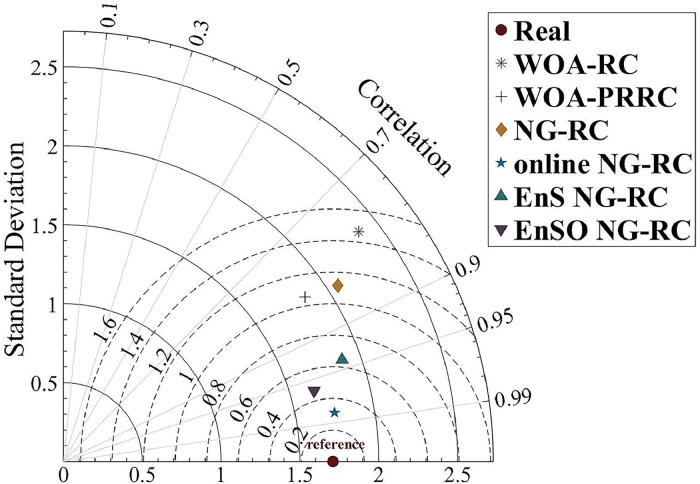
Taylor diagram of COD predicted values.

A Taylor diagram (Fig. 4) was constructed for the effluent COD to present the detailed prediction results. The Taylor diagram illustrates the relationships between standard deviation, centralized RMSE and correlation coefficients of six models relative to the observed values. It can be seen that online NG-RC, EnS NG-RC and EnSO NG-RC show superior performance across these three metrics compared to the other models.

**Fig. 5:**
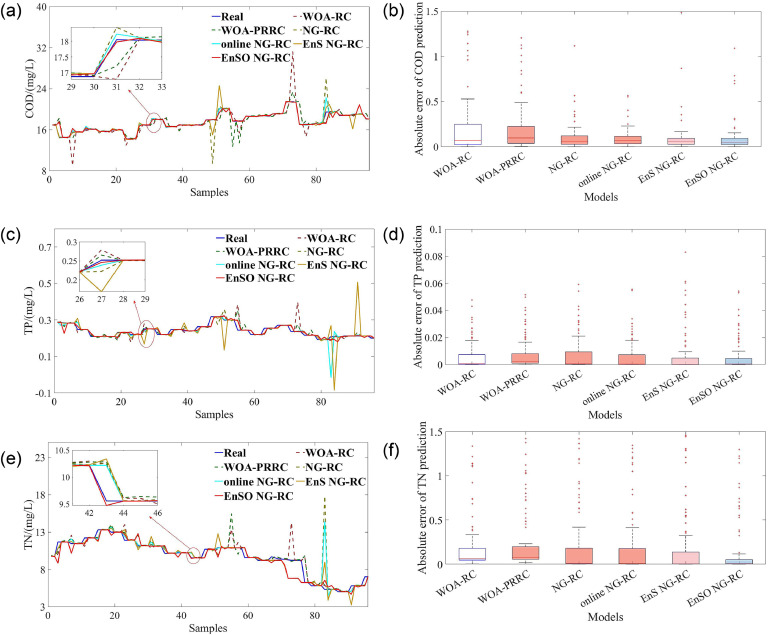
Fitting graphs of prediction results and box plots of absolute errors. (a), (c), and (e) represent the fitting graphs for COD, TP, and TN prediction results, respectively. (b), (d), and (f) illustrate the box plots of absolute residual errors for COD, TP, and TN predictions, respectively.

To comprehensively evaluate model performance, we constructed fit graphs and box plots of AE for the prediction results, as shown in Fig. 5. From Fig. 5(a), (c), and (e), we can see that the online NG-RC and EnSO NG-RC models exhibit superior tracking performance compared to NG-RC, particularly at peak and trough positions. This demonstrates the effectiveness of the proposed online learning strategy in enhancing the real-time tracking capabilities of NG-RC. Fig. 5(b), (d), and (f) illustrate that EnS NG-RC displays a more concentrated distribution of AE compared to NG-RC, indicating that the ensemble sparse identification algorithm reduces the sensitivity of NG-RC to noise and improves the stability of prediction results. Furthermore, the EnSO NG-RC, which integrates online learning and ensemble sparsification, balances tracking performance and residual concentration. This validates the efficacy of the proposed EnSO algorithm in enhancing real-time capabilities and noise resistance.

### 2.4 Comparison of running time for different models

This study selects NG-RC as the basis for soft sensor development due to its fewer hyperparameters, eliminating the need for time-consuming hyperparameter selection processes. Models based on NG-RC exhibit shorter response times than traditional RC, allowing for faster responses to changes in wastewater treatment processes. A comparative analysis of the running times for six models is presented in Supplementary Materials Table S4. The results show that NG-RC achieves the shortest runtime in both experiments. Additionally, online NG-RC, EnS NG-RC and EnSO NG-RC show significant advantages in terms of runtime compared to traditional RC, providing a robust basis for subsequent real-time monitoring applications.

## 3 Conclusions

This paper proposes a soft sensor based on NG-RC, termed EnSO NG-RC, to address the real-time monitoring problems of effluent qualities in WWTPs and act towards digital twins of effluent quality sensors. An incremental strategy is designed to enhance the online learning capability of NG-RC for incremental data blocks. Then the ensemble sparse strategy is combined for sparse identification of online-updated output weights, thereby reducing model overfitting issues. Furthermore, the effectiveness was validated using two real wastewater treatment datasets, where EnSO NG-RC achieved the smallest overall prediction error-RMSSD of 6.302 and 0.734, respectively. These values represent a reduction of 15.8 % and 58.1 % compared to the baseline NG-RC model. Additionally, these two WWTPs differ significantly in terms of equipment modernization and types of sewer systems, demonstrating the robustness and generalizability of our method. Future work will involve deploying the methodology in a broader range of actual WWTPs, followed by simulations to develop digital twins.

## 4 Materials and methods

### 4.1 Online next generation reservoir computing

NG-RC, proposed by Gauthier et al. (T.C. Wang et al., 2024), is a method mathematically equivalent to nonlinear vector autoregression. A detailed description of this approach is provided in the **Supplementary Materials Text S1**. NG-RC is a method characterized by offline training and online application. To adapt to the changing wastewater treatment process, encompassing weather conditions, seasonal variations and industrial activities, this paper proposes an online NG-RC model inspired by the Woodbury matrix identity (Hager, 1989). The model is designed to process continuously measured new data. First, we develop an incremental strategy that allows the model to update using previous networks and incorporate new samples without requiring a complete training cycle. The definition of this strategy is as follows:

(2) $$H^{k+1}=\left[H^{k},H_{1}\right],\;Y^{k+1}=\left[Y^{k},Y_{1}\right].$$

Note that $H^{k}$ and $H^{k+1}$ represent the feature vectors of the *k*-th and (*k* + 1)-th models, respectively, equivalent to $H_{total}$ mentioned in **Supplementary Materials Text S1**. $H_{1}$ and $Y_{1}$ denote the feature vector and system output corresponding to the newly added data.

Similar to the calculation of output weights in RC, NG-RC obtains the output weights $W_{out}$ by solving Eq. (3):

(3) $$\underset{W_{\text{out}}}{min}:{∥Y-W_{\text{out}}H∥}_{2}^{2}$$

(4) $$W_{\text{out}}=YH^{T}{\left(HH^{T}\right)}^{-1}$$

Next, we construct the Woodbury matrix identity to implement online updates for NG-RC. Assuming the initial training set is $R_{0}={\left(u_{k},y_{k}\right)}_{k=1}^{n_{0}}$, the output weights are:

(5) $$W_{out}^{0}=Y_{0}H_{0}^{T}T_{0}^{-1},\;\;T_{0}=H_{0}H_{0}^{T}$$

When a new data block $R_{1}={\left(u_{k},y_{k}\right)}_{k=n_{0}+1}^{n_{0}+n_{1}}$ is measured, the calculation of output weights is transformed into the following minimization problem:

(6) $$\underset{W_{out}^{1}}{min}:∥[Y_{0\;},Y_{1}]-W_{out}^{1}[H_{0\;},H_{1}]{∥}_{2}^{2}$$

By solving Eq. (6), we obtain $W_{out}^{1}$:

(7) $$W_{out}^{1}=\left[Y_{0\;},Y_{1}\right]{\left[H_{0\;},H_{1}\right]}^{T}T_{1}^{-1},\;\;T_{1}=\;T_{0}+H_{1}H_{1}^{T}$$

Where,

$$\left[Y_{0\;},Y_{1}\right]{\left[H_{0\;},H_{1}\right]}^{T}=Y_{0\;}H_{0}^{T}+Y_{1}H_{1}^{T}=Y_{0\;}H_{0}^{T}T_{0}^{-1}\;T_{0}+Y_{1}H_{1}^{T}=W_{out}^{0}\;T_{0}+Y_{1}H_{1}^{T}=W_{out}^{0}\left(\;T_{1}-H_{1}H_{1}^{T}\right)+Y_{1}H_{1}^{T}=W_{out}^{0}\;T_{1}-W_{out}^{0}H_{1}H_{1}^{T}+Y_{1}H_{1}^{T}$$

$$T_{1}^{-1}={\left(T_{0}+H_{1}\;H_{1}^{T}\right)}^{-1}=T_{0}^{-1}-T_{0}^{-1}H_{1}{\left(I+H_{1}^{T}\;T_{0}^{-1}\;H_{1}\right)}^{-1}H_{1}^{T}T_{0}^{-1}$$

Thus, we obtain $W_{out}^{1}$:

(8) $$W_{out}^{1}=W_{out}^{0}+\left(Y_{1}-W_{out}^{0}H_{1}\right)H_{1}^{T}T_{1}^{-1}$$

In summary, when the (*k* + 1)-th data block arrives, the recursive formula for output weights is:

(9) $$\Phi_{k+1}=\Phi_{k}-\Phi_{k}H_{k+1}{\left(I+H_{k+1}^{T}\;\Phi_{k}\;H_{k+1}\right)}^{-1}H_{k+1}^{T}\Phi_{k}$$

(10) $$W_{out}^{k+1}=W_{out}^{k}+\left(Y_{k+1}-W_{out}^{k}H_{k+1}\right)H_{k+1}^{T}\Phi_{k+1}$$

where, $\Phi_{k+1}=T_{k+1}^{-1}$.

Finally, this paper constructs a soft sensor based on online NG-RC, incorporating data preprocessing and auxiliary variable selection methods, for monitoring quality variables in WWTPs. The implementation process of this model is detailed in Algorithm 1.

**Algorithm 1:** Soft Sensor development based on online NG-RC.

**Input:** State variables: $\in R^{M\times n}$**,***M:* Number of auxiliary variables, *n*: Number of sample points; Initial training set:$\;R_{0}={\left(u_{k},y_{k}\right)}_{k=1}^{n_{0}}$, Incremental training set: $R_{1}={\left(u_{k},y_{k}\right)}_{k=n_{0}+1}^{n_{0}+n_{1}}$.
**Output:** Output variables: $\in R^{L\times n}$**,***L*: Number of target variables.
**Main steps:**
1) Pre-processing of date, including anomaly detection, standardization, and time difference. And determine the target variable y.
2) Auxiliary variable selection. Auxiliary variables are determined by combining domain knowledge with methods such as VIP and LASSO.
3) Build and train online NG-RC model
**If**$n\in R_{0}$, **then**
a. Construct the linear feature vector $H_{lin}$ by selecting input data from different time points.
b. Derive the nonlinear feature vector $H_{nlin}$ from $H_{lin}$.
c. Construct the total feature vector $H_{\text{total}}$ by combining $H_{lin}$ and $H_{nlin}$.
d. Solve the output matrix $W_{out}^{0}$ according to Eq. (5).
**elseif**$n\in R_{1}$**then**
Solve the output matrix $W_{out}^{1}$ according to (9), (10).
**endif**
4) The target output Y is obtained according to $Y=W_{\text{out}}H_{\text{total}}$.

### 4.2 Ensemble sparse next generation reservoir computing

Inspired by the principles of sparse identification of nonlinear dynamics (SINDy) (Brunton et al., 2016), this study considers the feature vector ***H***, composed of process variables and their delayed vectors, as the input ***X*** in the SINDy algorithm, while the time series matrix of quality variables is treated as the output $\overset{˙}{X}$. A detailed description of SINDy is provided in the **Supplementary Materials Text S3**. To mitigate overfitting issues, the output weights of the NG-RC are sparsified. However, the SINDy algorithm exhibits high sensitivity to noise during training, particularly when dealing with limited data. Therefore, building upon the SINDy algorithm, this paper introduces a bagging architecture into the sparse identification of NG-RC model, proposing an EnS NG-RC method. The bagging architecture, proposed by Breiman, is a parallel ensemble method based on bootstrap sampling (Breiman, 1996). Bagging resamples data with replacement, trains individual learners on each subset, and combines their outputs to obtain the final regression or classification results. This architecture reduces model sensitivity to data, prevents overfitting, and decreases model variance. It has been widely applied in various fields, including stock market prediction (Luo et al., 2023), traffic trajectory recognition (Zhai et al., 2024), and weather forecasting (Jayashree et al., 2023).

In this study, we first employ the bootstrap method to perform *q* iterations of bootstrap sampling, resulting in *q* subsets. Subsequently, each subset is trained individually, and the sequential thresholded least-squares (STLS) algorithm from the literature (Brunton et al., 2016) is applied to sparsify the system output weights. Finally, the outputs of the individual learners are combined, and the inclusion probabilities of each item are calculated and compared against a set threshold to determine the final output weights $W_{out}$. The implementation process for EnS NG-RC is detailed in Algorithm 2.

**Algorithm 2:** Ensemble sparse NG-RC.

**Input:** Feature vector: $\in R^{d\times n}$**,** SI: Sparse identification; *d:* Dimension of the total feature vector, *n*: Number of sample points; Output variables: $Y\in R^{L\times n}$**,***L*: Number of target variables. *p*: The highest order of the nonlinear function library. $\lambda_{1}$: Sparse threshold, $\lambda_{2}$: Regularization parameter, $\lambda_{3}$: Inclusion probability threshold. *q*: Number of bootstraps.
**Output:** Sparse output weights: $W_{out}$; Coefficient inclusion probabilities: $p_{ip}$.
**Main steps:**
1) Perform *q* iterations of bootstrap sampling on the original dataset to generate *q* subsets.
2) Build and train ensemble sparse NG-RC model for per subsample.
a. Solve the output matrix $W_{out}$ according to Eq. (4).
b. Sparsify $W_{out}$ utilizing the STLS algorithm.
**for**i∈{1,2,⋯,10}**do**
SI= ($abs\left(W_{out}\right)<\lambda_{1}$);
$\;\;\;W_{out}\left(SI\right)=0$;
**for** j=1:*L***do**
$\;\;\;\;b_{j}=∼SI\left(:,j\right)$;
Solve the output matrix $W_{out,j}\left(b_{j}\right)$ according to Eq. (4);
$\;\;\;\;W_{out}\left(:,j\right)=W_{out,j}\left(b_{j}\right)$;
**end**
**end**
3) Calculate the inclusion probabilities $p_{ip}$ for non-zero elements in $W_{out}$.
4) Perform ensemble sparsification on $W_{out}$ by setting elements with inclusion probabilities below $\lambda_{3}$ to zero, yielding the final output weights: $W_{out}\left(p_{ip}<\lambda_{3}\right)=0$.

Based on the above analysis, this paper constructs a soft sensor on the foundation of EnS NG-RC, integrating data preprocessing and auxiliary variable selection methods as outlined in Algorithm 1, to monitor quality variables in WWTPs.

### 4.3 Ensemble sparse online next generation reservoir computing

**Fig. 6:**
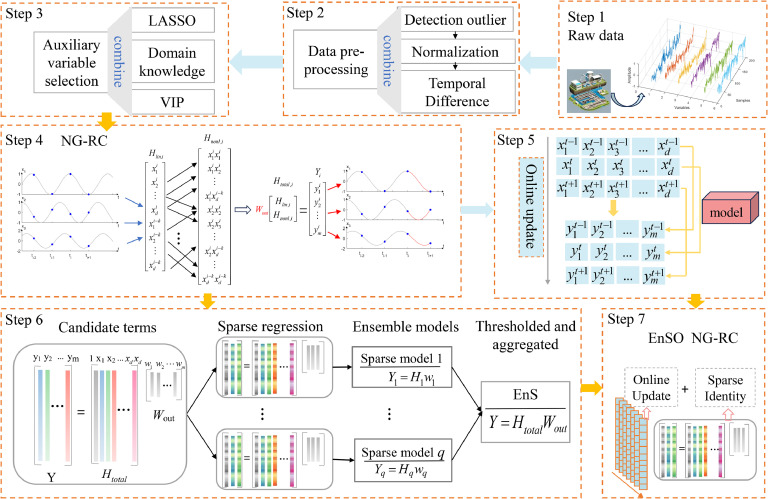
Diagram of EnSO NG-RC.

In this section, we propose a soft sensor, called EnSO NG-RC, to meet the real-time requirements of WWTPs while mitigating overfitting problems. Firstly, the collected sensor data are pre-processed, including outlier detection, normalization and time differencing. Secondly, auxiliary variables are selected using methods such as LASSO and VIP in conjunction with domain knowledge. Thirdly, the data is modeled and analyzed using the NG-RC method. Finally, online learning and ensemble sparsification are applied to the NG-RC framework, resulting in the final EnSO NG-RC soft sensor. The framework of this model is illustrated in Fig. 6.

## CRediT authorship contribution statement

**Gang Fang:** Writing – original draft, Software, Methodology. **Daoping Huang:** Supervision, Funding acquisition, Conceptualization. **Zhiying Wu:** Formal analysis, Data curation, Conceptualization. **Yan Chen:** Visualization, Formal analysis, Conceptualization. **Yan Li:** Supervision, Data curation, Conceptualization. **Yiqi Liu:** Writing – review & editing, Supervision, Funding acquisition.

## Declaration of competing interest

The authors declare that they have no known competing financial interests or personal relationships that could have appeared to influence the work reported in this paper.

## Data Availability

Data will be made available on request

## Appendix Supplementary materials

Image, application 1

Image, application 2

Image, application 3
