# Supplementary figures and images for: Effluent quality soft sensor for wastewater treatment plant with ensemble sparse learning-based online next generation reservoir computing
Source: Water Res X. 2024 Nov 10;25:100276. doi: 10.1016/j.wroa.2024.100276 (PMC11613165; doi:10.1016/j.wroa.2024.100276)

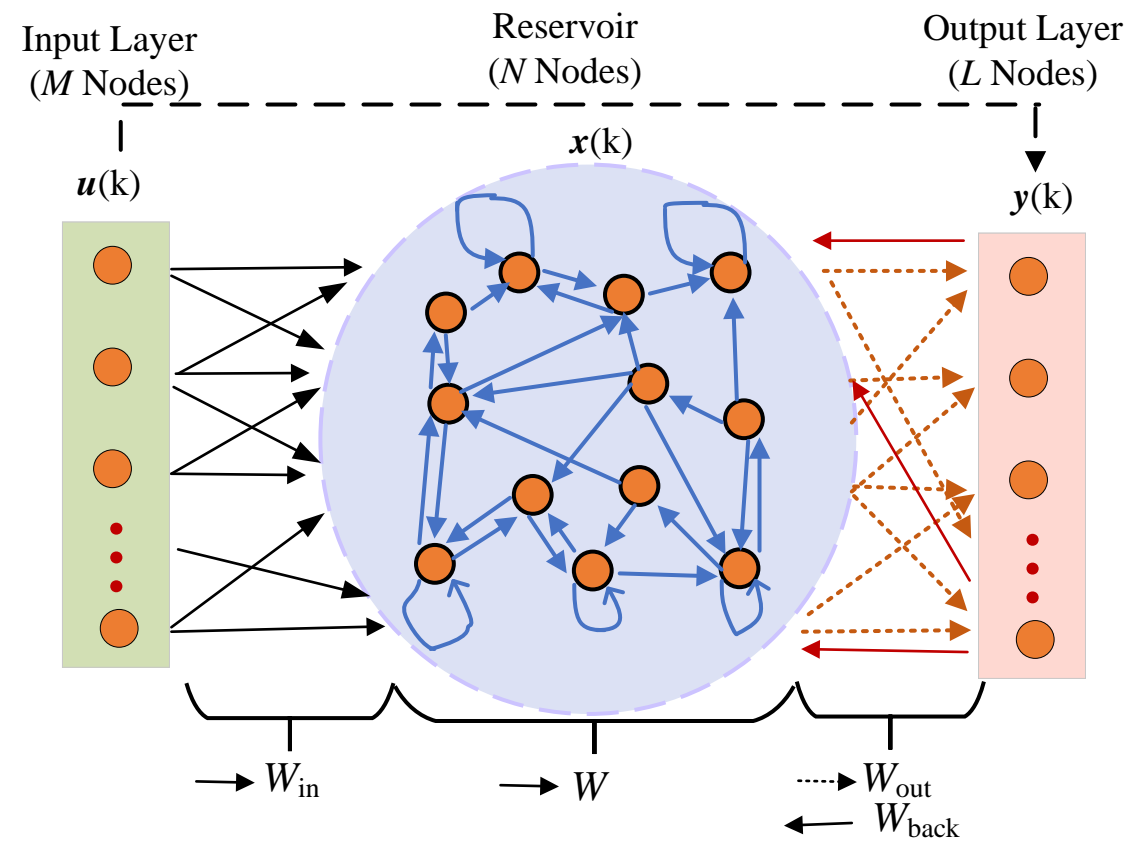

Supplement: Supplementary file 1 [file mmc1.pdf]

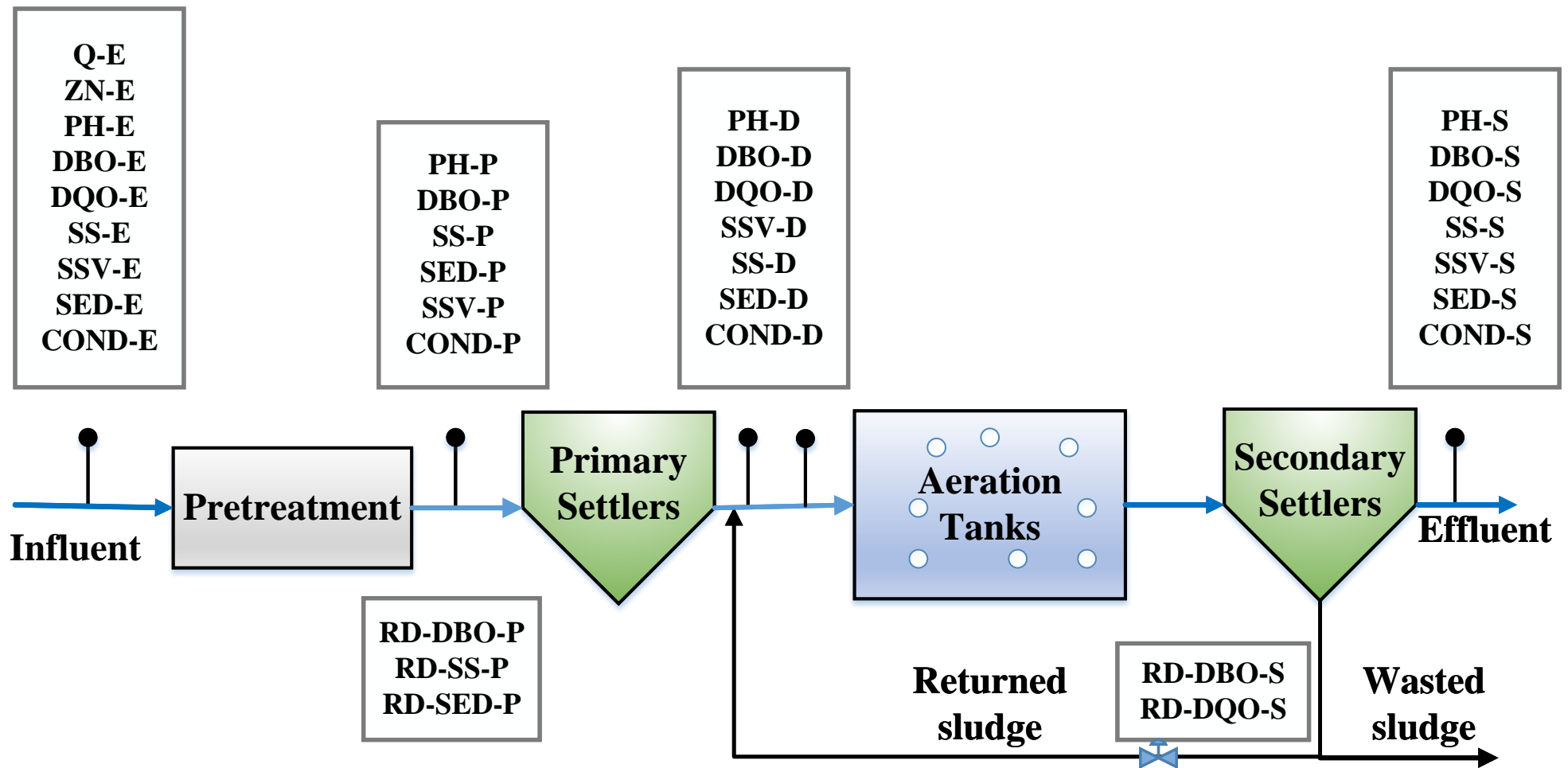

Supplement: Supplementary file 2 [file mmc2.pdf]
