# Supplementary material for: Effluent quality soft sensor for wastewater treatment plant with ensemble sparse learning-based online next generation reservoir computing
Source: Water Res X. 2024 Nov 10;25:100276. doi: 10.1016/j.wroa.2024.100276 (PMC11613165; doi:10.1016/j.wroa.2024.100276)
Supplement: Supplementary file 3 [file mmc3.pdf]

Supplementary Materials for

Effluent Quality Soft Sensor for Wastewater Treatment Plants with Ensemble Sparse Learning-Based Online Next Generation Reservoir Computing

The supplementary information consists of 6 pages, including 3 pieces of text, 4 tables and 2 figures.

Text S1. Next generation reservoir computing

Liquid state machines (LSM) and echo state networks (ESN) are two primary types of RC, with ESN being the most widely applied. In this paper, RC specifically refers to ESN. The structure of RC comprises an input layer, a reservoir layer, and an output layer (Fig. S1). At time k, the equations for updating the reservoir state and output of RC are given by Eq. (S1) and Eq. (S2), respectively.

$$\boldsymbol{x}(k+1) = \alpha f\left(\boldsymbol{W}_{in}\boldsymbol{u}(k+1) + \boldsymbol{W}\boldsymbol{x}(k) + \boldsymbol{W}_{back}\boldsymbol{y}(k)\right) + (1-\alpha)\boldsymbol{x}(k) \tag{S1}$$

$$y(k+1) = g(\boldsymbol{W}_{out}(\boldsymbol{u}(k+1), \boldsymbol{x}(k+1))) \tag{S2}$$

Where α represents the leak rate, while f and g denote the activation functions. The weight matrices $\boldsymbol{W}_{in}$, $\boldsymbol{W}$, $\boldsymbol{W}_{back}$ and $\boldsymbol{W}_{out}$ correspond to the input, reservoir internal, output feedback, and output weights, respectively. During the learning process, $\boldsymbol{W}_{in}$, $\boldsymbol{W}$ and $\boldsymbol{W}_{back}$ are randomly generated, and only $\boldsymbol{W}_{out}$ requires training. The output weights of RC are typically computed using the following equation:

$$\boldsymbol{W}_{out} = (\boldsymbol{X}^T\boldsymbol{X} + \lambda\boldsymbol{I})^{-1}\boldsymbol{X}^T\boldsymbol{Y} \tag{S3}$$

In which, $\boldsymbol{X}$ represents the matrix composed of reservoir state vectors, $\boldsymbol{Y}$ denotes the target output at corresponding time steps, λ is the regularization coefficient, and $\boldsymbol{I}$ represents the identity matrix.

Recent studies have shown that RC with nonlinear readout layers is mathematically equivalent to NVAR processes, both serving as equally powerful function approximators. This approach has been termed NG-RC in the literature. NG-RC replaces the reservoir in traditional RC with specifically constructed feature vectors. The feature vectors are composed of the input data at time k and its vectors delayed by j steps. The construction formula for these feature vectors is as follows:

$$\boldsymbol{H}_{lin,k} = \boldsymbol{x}_k \oplus \boldsymbol{x}_{k-s} \oplus \boldsymbol{x}_{k-2s} \oplus \cdots \oplus \boldsymbol{x}_{k-(j-1)s} \tag{S4}$$

$$\boldsymbol{H}_{\text{nlin}}^{(p)} = \boldsymbol{H}_{\text{lin}}\lceil\otimes\rceil\boldsymbol{H}_{\text{lin}}\lceil\otimes\rceil\cdots\lceil\otimes\rceil\boldsymbol{H}_{\text{lin}} \tag{S5}$$

$$\boldsymbol{H}_{\text{total},k} = c \oplus \boldsymbol{H}_{\text{lin},k} \oplus \boldsymbol{H}_{\text{nlin},k} \tag{S6}$$

$$\boldsymbol{Y}_k = \boldsymbol{W}_{\text{out}}\boldsymbol{H}_{\text{total},k} \tag{S7}$$

Where $\boldsymbol{H}_{lin,k}$ represents the linear component of the feature vector at time k, composed of the input vector $\boldsymbol{x}_k = \left[x_{1,k}, x_{2,k}, \cdots, x_{M,k}\right]^T$, j denotes the delay step and s is

the interval between steps. $\boldsymbol{H}_{nlin,k}$ represents the nonlinear component of the feature vector, with $\boldsymbol{H}_{\mathrm{nlin}}^{(p)}$ being the p-th order nonlinear polynomial feature vector, where the superscript p indicates that $\boldsymbol{H}_{nlin}$ appears p times. The symbol $\oplus$ denotes vector concatenation, $\otimes$ represents the outer product of vectors, and $\lceil\otimes\rceil$ is defined as the unique monomial operator that collects the results of the outer products. $\boldsymbol{H}_{\mathrm{total},k}$ denotes the total feature vector, c is a constant, and $\boldsymbol{Y}_k$ represents the system output. In contrast to traditional RC, NG-RC establishes direct connections from the data itself rather than utilizing pre-connected nodes as in RC, thereby reducing unnecessary connections and resulting in a more concise model.

Text S2. Woodbury matrix identity

The Woodbury matrix identity, also known as the matrix inversion lemma, greatly improves computational efficiency when solving large-scale matrix inversion problems. This paper proposes an online learning strategy based on the Woodbury matrix identity, which incorporates incremental samples for real-time updates. Assuming that $\boldsymbol{A}$, $\boldsymbol{C}$, $(\boldsymbol{A}+\boldsymbol{FCG})$ and $(\boldsymbol{C}^{-1}+\boldsymbol{GA}^{-1}\boldsymbol{F})$ are all invertible, where $\boldsymbol{A}\in\mathbb{R}^{\boldsymbol{a}\times\boldsymbol{a}}$, $\boldsymbol{F}\in\mathbb{R}^{\boldsymbol{a}\times\boldsymbol{b}}$, $\boldsymbol{C}\in\mathbb{R}^{\boldsymbol{b}\times\boldsymbol{b}}$ and $\boldsymbol{G}\in\mathbb{R}^{\boldsymbol{b}\times\boldsymbol{a}}$, the Woodbury matrix identity states:

$$(\boldsymbol{A}+\boldsymbol{FCG})^{-1}=\boldsymbol{A}^{-1}-\boldsymbol{A}^{-1}\boldsymbol{F}(\boldsymbol{C}^{-1}+\boldsymbol{GA}^{-1}\boldsymbol{F})^{-1}\boldsymbol{GA}^{-1} \tag{S8}$$

In special cases, Eq. (S8) can be transformed into the following two forms:

1) When $\boldsymbol{C}$ is the identity matrix $\boldsymbol{I}\in\mathbb{R}^{\boldsymbol{b}\times\boldsymbol{b}}$:

$$(\boldsymbol{A}+\boldsymbol{FG})^{-1}=\boldsymbol{A}^{-1}-\boldsymbol{A}^{-1}\boldsymbol{F}(\boldsymbol{I}+\boldsymbol{GA}^{-1}\boldsymbol{F})^{-1}\boldsymbol{GA}^{-1} \tag{S9}$$

2) Consider two column vectors $\boldsymbol{c},\boldsymbol{g}\in\mathbb{R}^{\boldsymbol{a}}$, with the condition that $1+\boldsymbol{g}^{\boldsymbol{T}}\boldsymbol{A}^{-1}\boldsymbol{c}\neq 0$. This leads to the Sherman-Morrison formula:

$$(\boldsymbol{A}+\boldsymbol{cg}^{T})^{-1}=\boldsymbol{A}^{-1}-\frac{\boldsymbol{A}^{-1}\boldsymbol{cg}^{T}\boldsymbol{A}^{-1}}{1+\boldsymbol{g}^{T}\boldsymbol{A}^{-1}\boldsymbol{c}} \tag{S10}$$

Text S3. Sparse identification of dynamic systems

The sparse identification of nonlinear dynamics (SINDy) algorithm, proposed by Brunton et al., is a novel method for sparse identification of nonlinear dynamical systems. SINDy posits that most dynamical systems can be defined by a few relevant terms and employs sequential thresholded least-squares (STLS) for sparse identification. The algorithm identifies nonlinear governing equations from TS data, with the dynamical state of the system evolving over time. This evolution can typically be represented as:

$$\frac{dx(k)}{dk}=f(x(k)) \tag{S11}$$

Where dx/dk represents the derivative of the target variable with respect to time, $x(k)$ denotes the state of the system at time k, and $f(x(k))$ represents the dynamic equation of the system. The steps for performing sparse identification on dynamic systems are as follows:

1) Collect state data from the system. Arrange M parameter variables and their

corresponding n state data into a TS matrix $\boldsymbol{X}$. Measure or calculate the derivative matrix $\dot{X}$ of the state variables, as shown in the following equation:

$$\boldsymbol{X} = \begin{bmatrix} \boldsymbol{x}_1{}^T \\ \boldsymbol{x}_2{}^T \\ \vdots \\ \boldsymbol{x}_n{}^T \end{bmatrix} = \begin{bmatrix} x_{1,1} & x_{2,1} & \cdots & x_{M,1} \\ x_{1,2} & x_{2,2} & & x_{M,2} \\ \vdots & \vdots & \ddots & \vdots \\ x_{1,n} & x_{2,n} & \cdots & x_{M,n} \end{bmatrix} \tag{S12}$$

$$\dot{\boldsymbol{X}} = \begin{bmatrix} \dot{\boldsymbol{x}}_1{}^T \\ \dot{\boldsymbol{x}}_2{}^T \\ \vdots \\ \dot{\boldsymbol{x}}_n{}^T \end{bmatrix} = \begin{bmatrix} \dot{x}_{1,1} & \dot{x}_{2,1} & \cdots & \dot{x}_{M,1} \\ \dot{x}_{1,2} & \dot{x}_{2,2} & & \dot{x}_{M,2} \\ \vdots & \vdots & \ddots & \vdots \\ \dot{x}_{1,n} & \dot{x}_{2,n} & \cdots & \dot{x}_{M,n} \end{bmatrix} \tag{S13}$$

2) Perform mathematical transformations such as power function, trigonometric, and logarithmic transformations on the parameters in $\boldsymbol{X}$. The resulting functions are termed basis functions, which are then combined to form a basis function library $H(\boldsymbol{X})$.

$$H(\boldsymbol{X}) = [1\ \boldsymbol{X}\ \boldsymbol{X}^2\ \cdots \sin(\boldsymbol{X}) \cos(\boldsymbol{X})\ \cdots] \tag{S14}$$

After determining $\dot{\boldsymbol{X}}$ and $H(\boldsymbol{X})$, express Eq. (S11) in vector form as follows:

$$\dot{\boldsymbol{X}} = H(\boldsymbol{X})\boldsymbol{\Gamma} \tag{S15}$$

3) Determine the coefficient vector $\boldsymbol{\Gamma} = [\gamma_1\ \gamma_2\ \cdots\ \gamma_n]$ for the basis functions, employing sparse regression to set the coefficients of relatively inactive basis functions to zero. Once $\boldsymbol{\Gamma}$ is established, the control equation at time k is given as follows:

$$\dot{\boldsymbol{x}}_k = f_k(\boldsymbol{x}) = H(\boldsymbol{x}^T)\gamma_k \tag{S16}$$

Therefore,

$$\dot{\boldsymbol{x}} = f(\boldsymbol{x}) = \boldsymbol{\Gamma}^T(H(\boldsymbol{x}^T))^T \tag{S17}$$

Table S1 The value of the hyperparameter.

Hyperparameters	Values
The highest order of the nonlinear function library	p =2
The number of delay steps of the feature vector	j=4
Sparse threshold	$\lambda_1 = 0.1$
Regularization parameter	$\lambda_2 = 0.05$
Inclusion probability threshold	$\lambda_3 = 0.5$
Number of bootstraps	q=150

Table S2 The value of the hyperparameter.

Hyperparameters	Values
The highest order of the nonlinear function library	p =2
The number of delay steps of the feature vector	j=3
Sparse threshold	$\lambda_1 = 0.1$
Regularization parameter	$\lambda_2 = 0.05$
Inclusion probability threshold	$\lambda_3 = 0.5$
Number of bootstraps	q=150

Table S3 Comparison of prediction results of six models of Dongguan dataset.

Model	RMSE			PCC			RMSSD
	COD	TP	TN	COD	TP	TN	
WOA-RC	1.466	0.025	0.805	0.789	0.765	0.945	1.673
WOA-PRRC	1.055	0.018	0.727	0.828	0.856	0.956	1.282
NG-RC	1.115	0.027	1.349	0.842	0.742	0.861	1.750
Online NG-RC	0.313	0.026	1.002	0.984	0.756	0.919	1.050
EnS NG-RC	0.648	0.049	0.666	0.939	0.457	0.965	0.930
EnSO NG-RC	**0.464**	**0.014**	**0.568**	**0.962**	**0.904**	**0.973**	**0.734**

Table S4 Running time of six models (seconds)

Model	Experiment I	Experiment II
NG-RC	0.173	0.302
Online NG-RC	0.247	0.521
EnS NG-RC	2.008	6.287
EnSO NG-RC	12.533	18.226
WOA-RC	84.515	95.431
WOA-PRESN	88.154	99.037

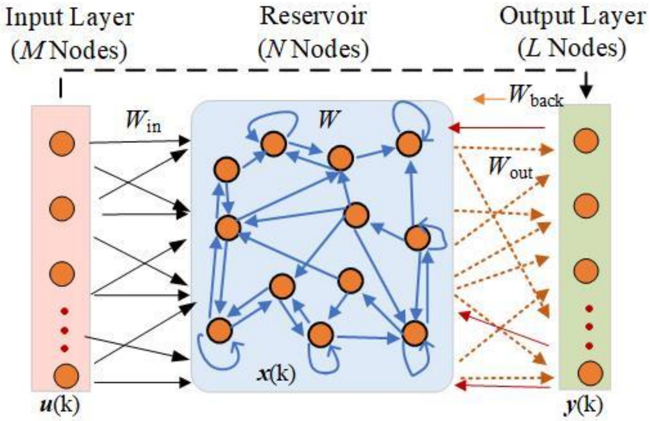

Fig.S1 The structure of the reservoir computing.

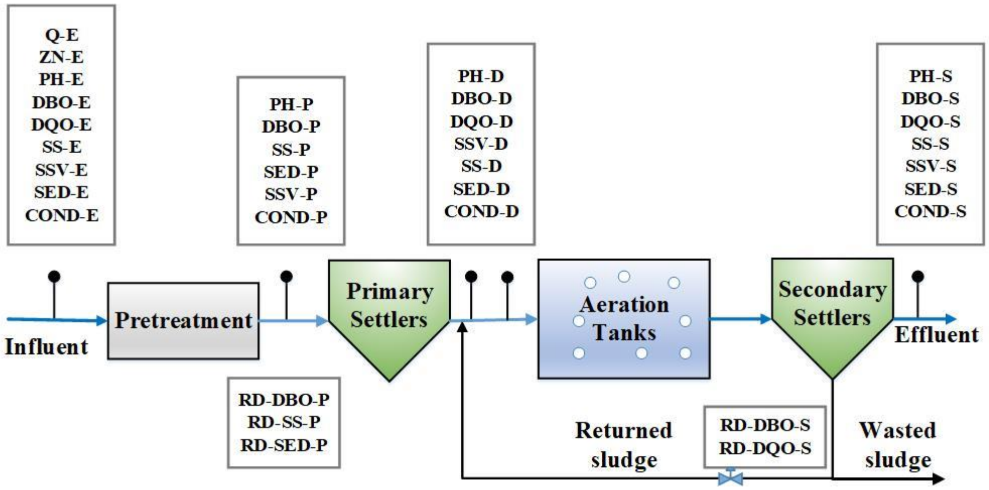

Fig.S2 The schematic of a Barcelona wastewater plant.
